# Supplementary material for: Student’s Inventory of Professionalism (SIP): A Tool to Assess Attitudes towards Professional Development Based on Palliative Care Undergraduate Education
Source: Int J Environ Res Public Health. 2019 Dec 5;16(24):4925. doi: 10.3390/ijerph16244925 (PMC6950691; doi:10.3390/ijerph16244925)
Supplement: Supplementary file 1 [file ijerph-16-04925-s001.pdf]

Table S1. Annex. INVENTARIO SIP

Las siguientes afirmaciones se refieren a **cómo valora usted en el momento actual su propio nivel de preparación a la hora de acercarse al paciente, relacionarse con sus colegas, y reflexionar sobre su trabajo**. Indique, por favor, su **grado de acuerdo** con las afirmaciones, marcando con un círculo el número entre 0 y 10 que le parezca adecuado (donde 0 es no estar nada de acuerdo y 10 estar totalmente de acuerdo).

Sub-escala	#	Item	Punctuación											Total
			Desacuerdo						*Acuerdo*					
**Cuidado holístico**	1	He aprendido a escuchar a los pacientes	0	1	2	3	4	5	6	7	8	9	10	
	2	He aprendido a mostrar mi humanidad al paciente	0	1	2	3	4	5	6	7	8	9	10	
	3	He aprendido a emplear con cada paciente el tiempo que necesita	0	1	2	3	4	5	6	7	8	9	10	
	4	He aprendido a dar soporte emocional y espiritual a mis pacientes	0	1	2	3	4	5	6	7	8	9	10	
	5	Me veo capaz de adaptarme a cada paciente	0	1	2	3	4	5	6	7	8	9	10	
	6	Me veo capaz de dar esperanza al paciente, sin crear falsas expectativas cuando hablo sobre el avance de la enfermedad	0	1	2	3	4	5	6	7	8	9	10	H:
	7	Me veo capaz de ayudar al paciente a preservar su dignidad aunque esté deteriorado	0	1	2	3	4	5	6	7	8	9	10	
	8	Me veo capaz de atender a la familia de mis pacientes y dar soporte a sus necesidades	0	1	2	3	4	5	6	7	8	9	10	
	9	He aprendido a ganarme la confianza del paciente	0	1	2	3	4	5	6	7	8	9	10	
	10	He aprendido a dar malas noticias a los pacientes	0	1	2	3	4	5	6	7	8	9	10	
	11	Me veo capaz de manejar correctamente mis emociones al tratar a los pacientes en situaciones complejas	0	1	2	3	4	5	6	7	8	9	10	
**Cuidar y Comprender**	12	He aprendido que cuidar es la esencia de mi profesión	0	1	2	3	4	5	6	7	8	9	10	
	13	He aprendido a ser cercano con los pacientes	0	1	2	3	4	5	6	7	8	9	10	
	14	He aprendido que ante una enfermedad con mal pronóstico/incurable, siempre se puede hacer algo	0	1	2	3	4	5	6	7	8	9	10	
	15	He aprendido a tener en cuenta las experiencias previas del paciente para poder comprenderle	0	1	2	3	4	5	6	7	8	9	10	CC:
	16	La experiencia clínica me ha ayudado a comprender cómo el enfermo se adapta a su enfermedad	0	1	2	3	4	5	6	7	8	9	10	
	17	Me veo capaz de escuchar a otros antes de tomar una decisión difícil	0	1	2	3	4	5	6	7	8	9	10	
**Crecimiento personal**	18	La experiencia clínica me ha ayudado a crecer como persona	0	1	2	3	4	5	6	7	8	9	10	
	19	La experiencia clínica me ha ayudado a comprender mejor a mis colegas de profesión	0	1	2	3	4	5	6	7	8	9	10	
	20	La experiencia clínica me ha ayudado a mantener o recuperar la ilusión en mi desarrollo como profesional de la salud	0	1	2	3	4	5	6	7	8	9	10	CP:
	21	He aprendido a valorar la gratitud del paciente	0	1	2	3	4	5	6	7	8	9	10	
**Trabajo en equipo**	22	Me veo capaz de trabajar en equipo	0	1	2	3	4	5	6	7	8	9	10	
	23	He aprendido a pedir consejo a mis colegas cuando es necesario	0	1	2	3	4	5	6	7	8	9	10	TE:
	24	He aprendido a buscar ayuda cuando la necesito	0	1	2	3	4	5	6	7	8	9	10	
**Toma de decisiones**	25	Me veo capaz de involucrar al paciente y a su familia en la toma de decisiones en situaciones complejas	0	1	2	3	4	5	6	7	8	9	10	TD:

	26	Reevalúo mi plan terapéutico cuando provoco sufrimiento	0	1	2	3	4	5	6	7	8	9	10	
	27	He aprendido a tener una actitud abierta, no juzgando al paciente por la primera impresión	0	1	2	3	4	5	6	7	8	9	10	
	28	He aprendido a respetar los deseos del paciente	0	1	2	3	4	5	6	7	8	9	10	
	29	He aprendido a individualizar la toma de decisiones	0	1	2	3	4	5	6	7	8	9	10	
	30	He aprendido que cada tratamiento ha de ser personalizado	0	1	2	3	4	5	6	7	8	9	10	
**Valoración del paciente**	31	Me veo capaz de realizar una correcta evaluación de síntomas	0	1	2	3	4	5	6	7	8	9	10	V:
	32	Me veo capaz de realizar una evaluación integral y multidimensional de los pacientes	0	1	2	3	4	5	6	7	8	9	10	
**Ser profesional de la salud**	33	He aprendido que mi profesión es ciencia con compasión	0	1	2	3	4	5	6	7	8	9	10	P:
**PUNTUACIÓN TOTAL**														________
